# Author Correction: Viral entry shapes HCMV latency establishment

**DOI:** 10.1038/s41467-026-73127-8

**Published:** 2026-05-18

**Authors:** Yaarit Kitsberg, Aharon Nachshon, Tamar Arazi, Karin Broennimann, Tal Fisher, Alexander Wainstein, Yaara Finkel, Noam Stern-Ginossar, Michal Schwartz

**Affiliations:** 1 Department of Molecular Genetics, Weizmann Institute of Science, Rehovot, Israel; 2 Present Address: Department of Molecular Cell Biology, Weizmann Institute of Science, Rehovot, Israel; 3 Present Address: Department of Bioengineering, Stanford University School of Medicine, Stanford, CA USA

**Keywords:** Herpes virus, Virus-host interactions, Virology

Correction to: *Nature Communications* 10.1038/s41467-025-68063-y, published online 29 December 2025

In the original version of this Article, Figure S8 in the Supplementary Information file was inadvertently cropped.

This has now been corrected in both the PDF and HTML versions of the Article.

