# Supplementary material for: Viral entry shapes HCMV latency establishment
Source: Nat Commun. 2025 Dec 29;17:1300. doi: 10.1038/s41467-025-68063-y (PMC12868673; doi:10.1038/s41467-025-68063-y)
Supplement: Supplementary file 1 — Supplementary Information [file 41467_2025_68063_MOESM1_ESM.pdf]

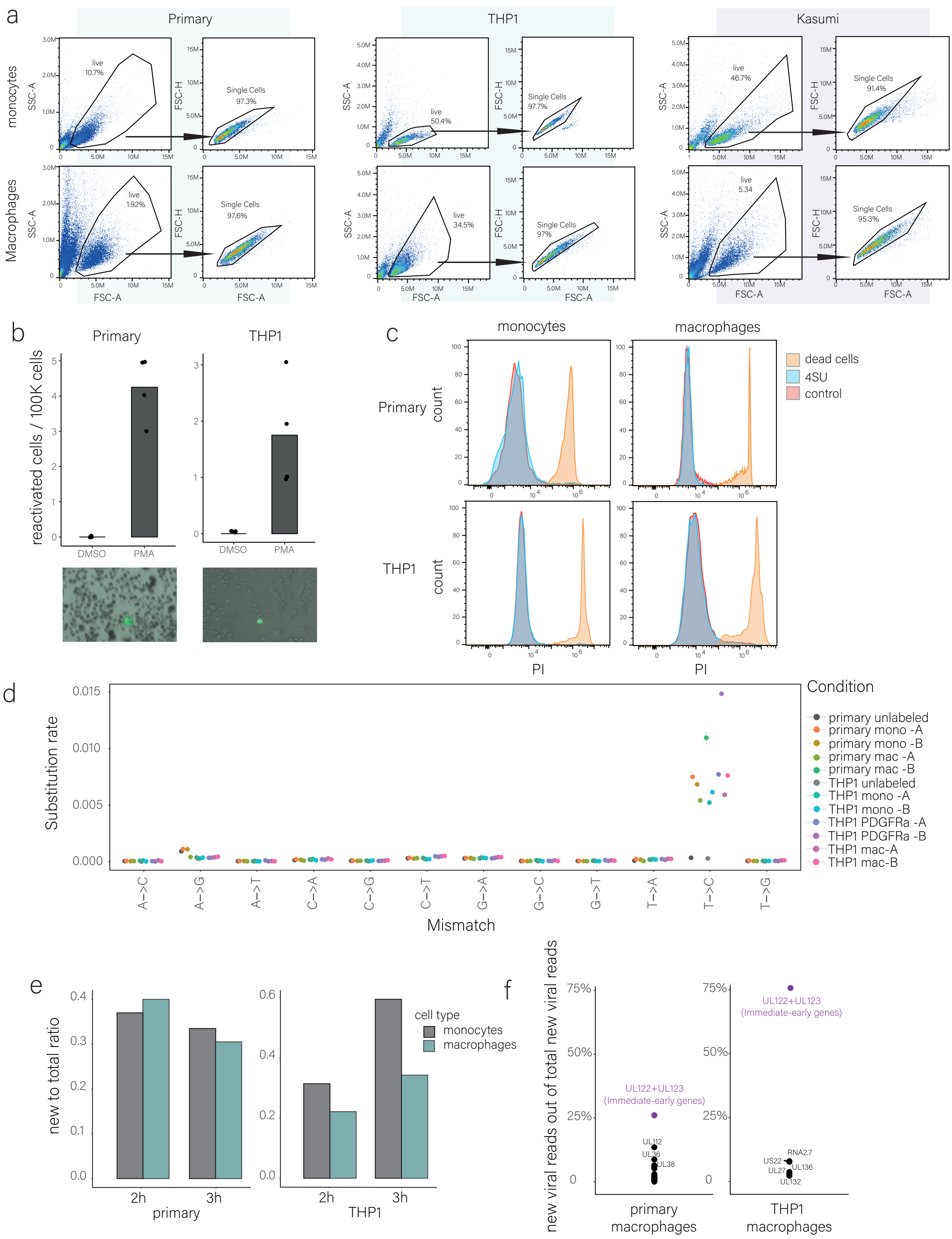

Fig. S1. HCMV infection and viral gene expression in monocytes and macrophages. (a). Gating strategy used to determine the single-cell population for all flow cytometry analyses of monocytes and macrophages. Gating strategy of the primary cells corresponds to figures 1b, 5c, 5d, 6b, 7b, 7c, S1c, S4a and S7a. Gating strategy of THP1 corresponds to figures 1b, 2b, 4a, 4b, 5b, 6b-d, 7b-d, 7g, 8b, 8d, 8f, S1c, S2d, S4a, S4b, S4h, S4k, S4l, S4n, S5a, S5c, S5e, S6a,S7a-c, S7e, S7f and S7g-l. Gating strategy of Kasumi corresponds to figure 1b. (b). Primary and THP1 monocytes were infected with HCMV-GFP. At 7 dpi, cells were treated with either DMSO or PMA. 3 days post PMA treatment, amount of GFP positive monocytes were counted. (c). Survival assay of primary and THP-1 monocytes and macrophages. Cells were treated with 4-thiouridine (4sU) or UPW as control for 3 hours, followed by propidium iodide (PI) staining and flow cytometry analysis to assess cell viability. PI-positive cells represent non-viable populations, boiled cells were used to mark the dead cell population. (d). Rates of nucleotide substitutions demonstrate efficient conversion rates in 4sU-treated samples compared to unlabeled cells (no 4sU, gray dots) (e). Proportion of new-to-total RNA of cellular transcripts in infected primary and THP1 monocytes and macrophages. Infected cells were labeled with 4sU at 3 hpi and collected for RNAseq after two (left bars) or three (adjacent right bars) hours post labeling. (f). Percentage of new viral gene reads out of total new viral reads in infected primary and THP1 macrophages at three hours post labeling (6 hpi).

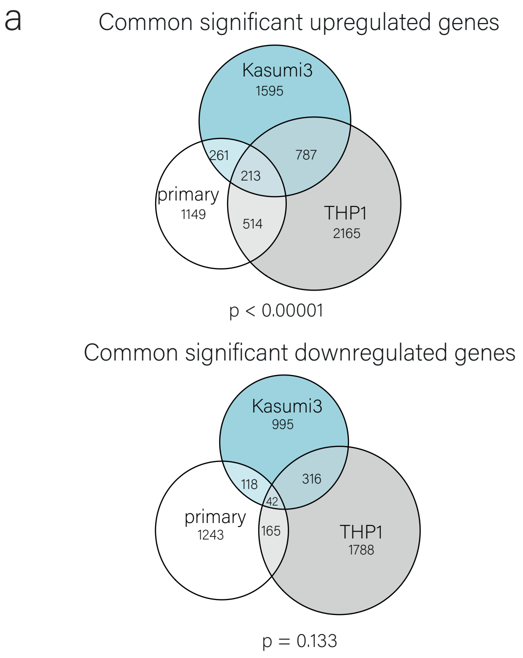

b

Gene Set Name	p-value
HALLMARK_IL2_STAT5_SIGNALING	1.70E-05
HALLMARK_TNFA_SIGNALING_VIA_NFKB	0.00015312
HALLMARK_KRAS_SIGNALING_UP	0.0013335
HALLMARK_TGF_BETA_SIGNALING	0.00204652

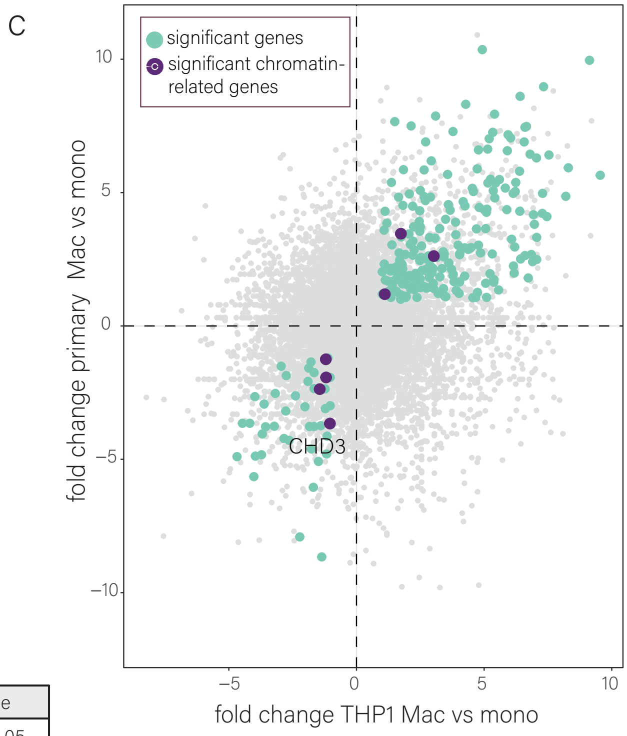

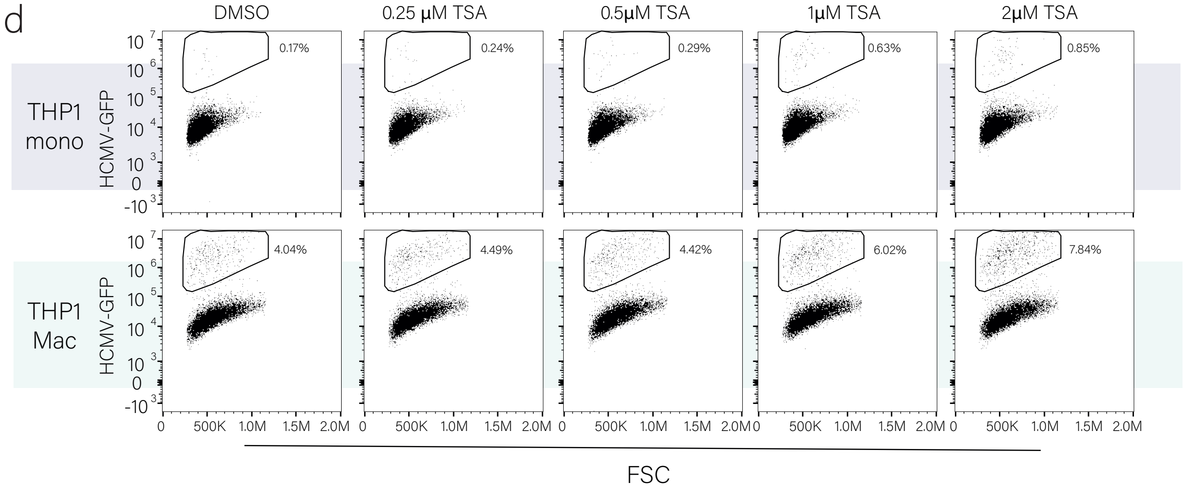

Fig. S2. Changes in gene expression upon differentiation. (a). Venn diagram summarizing the amount of significant upregulated and downregulated genes in all three cell types. Statistics on the common upregulated and downregulated genes were performed using hypergeometric test. (b). Hallmark pathway enrichment analysis shows four significantly enriched pathways in the 213 common significant upregulated genes. Analysis was performed using a hypergeometric test. (c). Scatterplot of the fold change (FC) from RNA-seq data between primary monocytes and macrophages, relative to the fold change between THP1 monocytes and macrophages. Light blue dots represent significantly upregulated genes in all three cell types. Dark purple dots represent chromatin-related genes that are significantly changing upon differentiation in all three cell types (p = 0.885 for the downregulated and p = 0.999 for the upregulated genes using a hypergeometric test), based on the EpiFactors database [53]. (d). Flow cytometry analysis of infected THP1 monocytes and macrophages, treated with TSA or DMSO as control at 5 hpi. Analysis was performed at 3 dpi. The gate marks the productive, GFP-bright cell population. Gating strategy is shown in Fig. S1a.

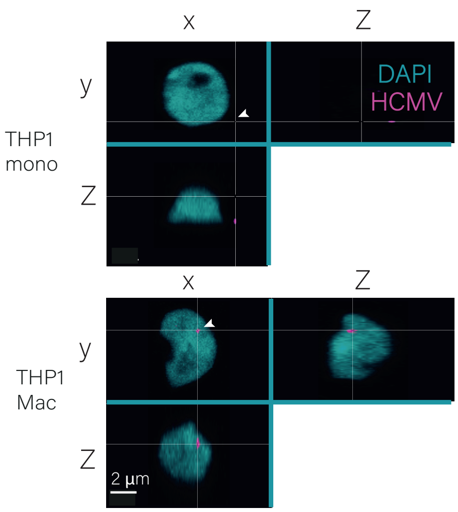

Fig. S3. **DNA-FISH on viral genomes.** 3D images of infected THP1 monocyte and macrophage nuclei at 12 hpi The HCMV genome was probed using 3D DNA-FISH

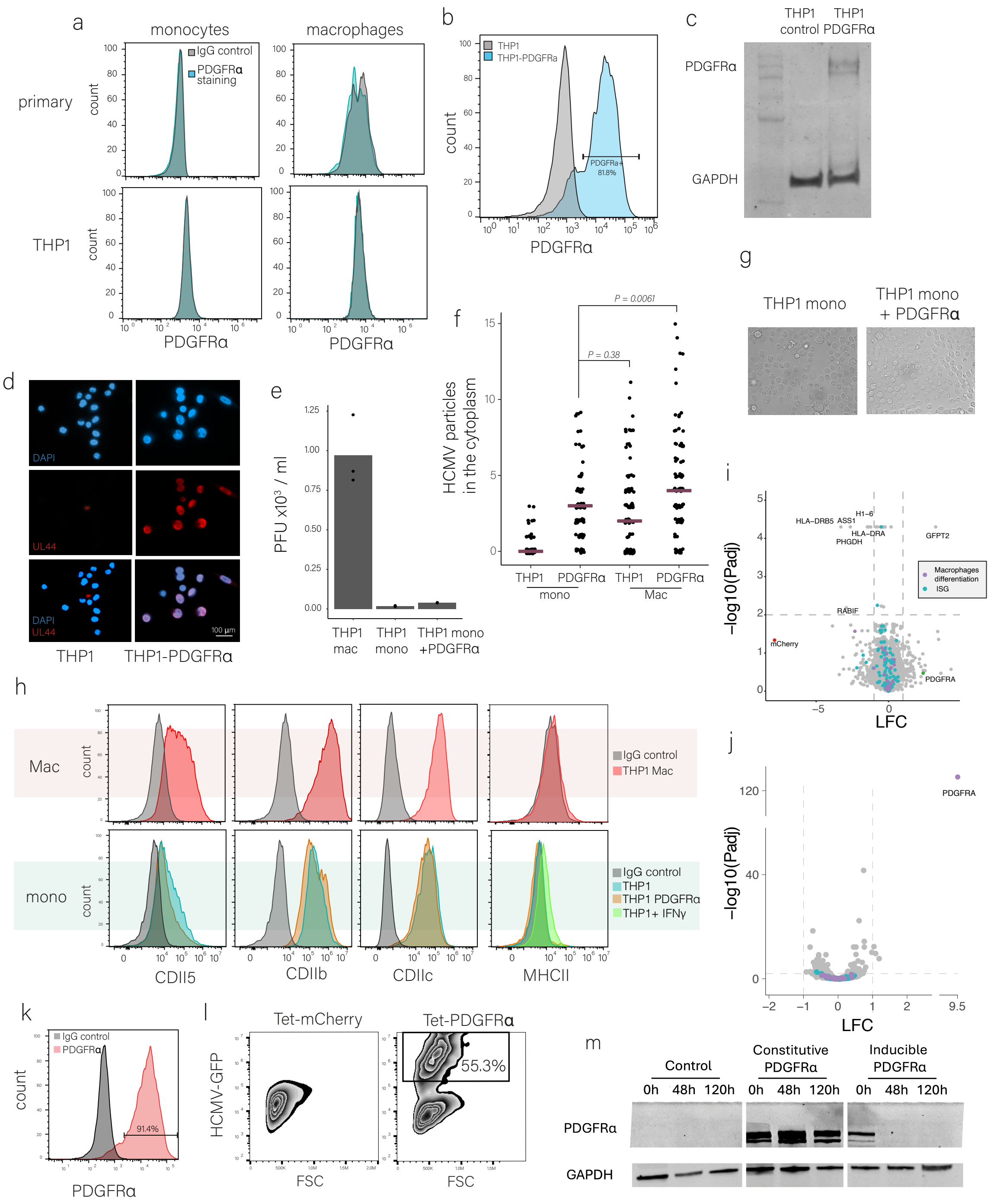

Fig. S4. Ectopic expression of PDGFRα leads to productive infection in monocytes. (a). Flow cytometry analysis of PDGFRα surface staining in primary and THP1 monocytes and macrophages. (b). Flow cytometry analysis of PDGFRα surface expression on THP1 and THP1–PDGFRα monocytes. (c). Western blot of THP1-PDGFRα versus control. GAPDH was used as loading control. (d). Infected THP1 and THP1–PDGFRα monocytes were stained for UL44 (replication compartments) at 4 dpi, and imaged by fluorescence microscopy. (e). THP1 macrophages, monocytes, and THP1-PDGFRα were infected with HCMV-GFP. At 7 dpi, supernatants were transferred onto fibroblasts, and GFP-positive fibroblasts were quantified at 2 dpi by flow cytometry to calculate PFU. n =2- 3 biological replicates. (f). Quantification of cytoplasmic viral particles of infected THP1 and THP1-PDGFRα monocytes or macrophages using HCMV-UL32-GFP at 1 hpi. (n Mac-PDGFRα=89, n Mac = 103, n mono =83, n mono-PDGFRα = 84). Viral particles were counted using FIJI image processing and statistics was performed using Poisson regression. (g). Light microscopy of THP1 and THP1-PDGFRα monocytes. (h). Flow cytometry analysis of differentiation surface markers on THP1 and THP1–PDGFRα monocytes and THP1 macrophages. For MHCII staining, monocytes treated with IFNγ are shown as positive control. (i-j). Differential analysis of Mass Spectrometry (MS) (i) or RNA-seq (j) from THP1 and THP1–PDGFRα monocytes. Blue dots represent innate-immune-related genes (ISGs, compiled based on [54]) and purple dots represent monocytes to macrophage differentiation-related genes (GO:0030225). n=3 for (i) and n=2 for (j). (k). Flow cytometry analysis of induced PDGFRα expression in THP1 monocytes 24 h post doxycycline treatment. (l). Flow cytometry analysis of THP1 monocytes and THP1 monocytes with induced expression of control or PDGFRα, infected with HCMV–GFP at 3 dpi. (m). Western blot of THP1 monocytes overexpressing control, constitutive or inducible or PDGFRα at 0, 48 and 120 hpi. GAPDH was used as loading control. Gating strategies for figures S4a, b, h, k and l are shown in Fig. S1a. Source data for figures S4e and f are provided as a Source Data file.

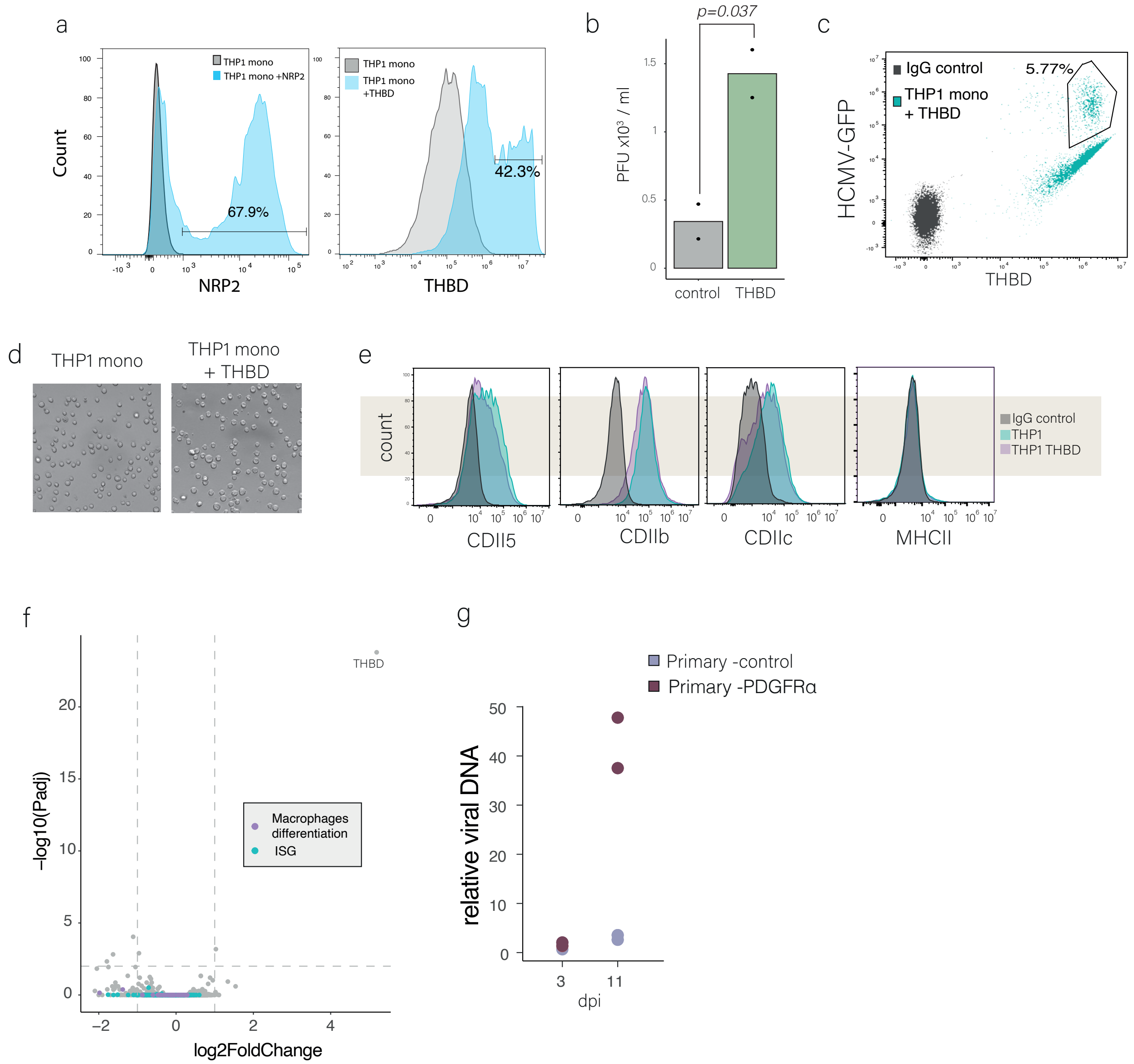

Fig. S5. PDGFRα and THBD expression facilitate productive infection. (a). Flow cytometry analysis of NRP2 or THBD surface expression in THP-1 monocytes with induced expression of NRP2 or THBD, respectively, compared to the parental cells. (b). THBD and control cells were infected with HCMV-GFP. At 8 dpi, viral supernatant was used to infect recipient wild-type fibroblasts. Forty-eight hours later, the percentage of GFP-positive recipient cells was determined by flow cytometry and used to calculate the number of plaque-forming units (PFU). n = 2. (c). Flow cytometry analysis of THP1 overexpressing THBD, infected with HCMV–GFP at 3 dpi showing HCMV-GFP level versus THBD surface level. (d). Light microscopy of THP1 monocytes overexpressing mCherry control or THBD. (e). Flow cytometry analysis of cell surface staining of differentiation markers on THP-1 and THP-1–THBD monocytes. Macrophages used as control are shown in Fig. S4h. For MHCII staining, monocytes treated with IFNγ are shown as positive control in Fig. S4h. (f). Differential gene expression analysis of RNA-seq data from THP1 mono overexpressing mCherry control or THBD. Blue dots represent genes related to innate immunity (Interferon Stimulated Genes, ISGs, compiled based on [54]) and purple dots represent genes related to monocytes to macrophage differentiation (GO:0030225). (g). Relative viral DNA in infected primary monocytes overexpressing PDGFRα at 3 and 11 dpi. n=2. Source data for figures S5b and g are provided as a Source Data file. Gating strategies of figures S5a, c and e are shown in Fig. S1a.

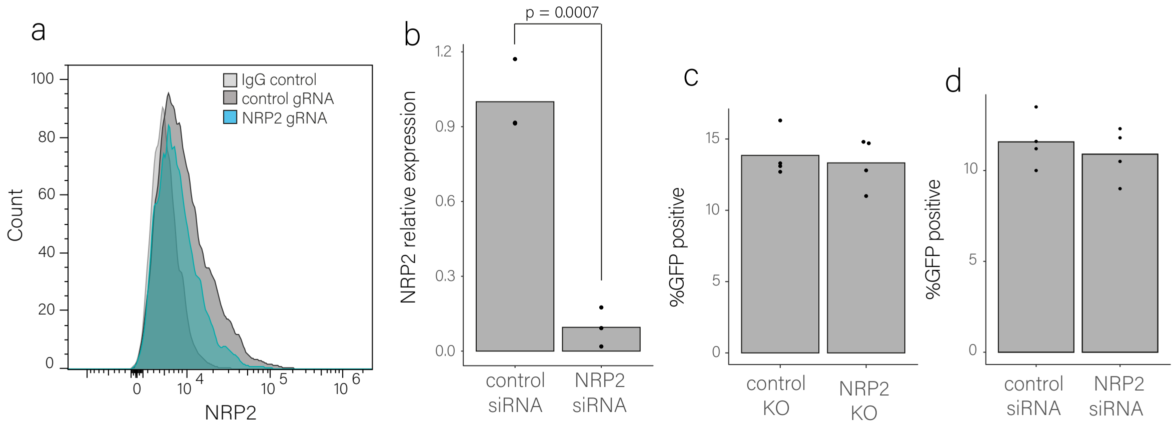

Fig. S6. NRP2 does not facilitate HCMV entry into macrophages. (a). Cell surface staining with NRP2 or IgG control of THP1 macrophages treated with CRISPR knockout against NRP2 or control. Cells were analyzed by Flow cytometry. Gating strategy is shown in Fig. S1a. (b). Relative expression level of NRP2, measured by qPCR in THP1 macrophages treated with NRP2 siRNA compared to control siRNA at the time of HCMV infection. p-value was calculated using a two-sided student t-test. n=3. (c). Quantification of flow cytometry analysis of THP1 macrophages with NRP2 or control CRISPR knockout, infected with HCMV-GFP. Analysis was performed at 3 dpi, p-value was calculated using a two-sided student t-test. n=4. (d). Quantification of flow cytometry analysis of THP1 macrophages, transfected with NRP2 and control siRNA two days before infection with HCMV-GFP. Analysis was performed at 3 dpi, p-value was calculated using a two-sided student t-test. n=4. Source data for figures S6b-d are provided as a Source Data file.

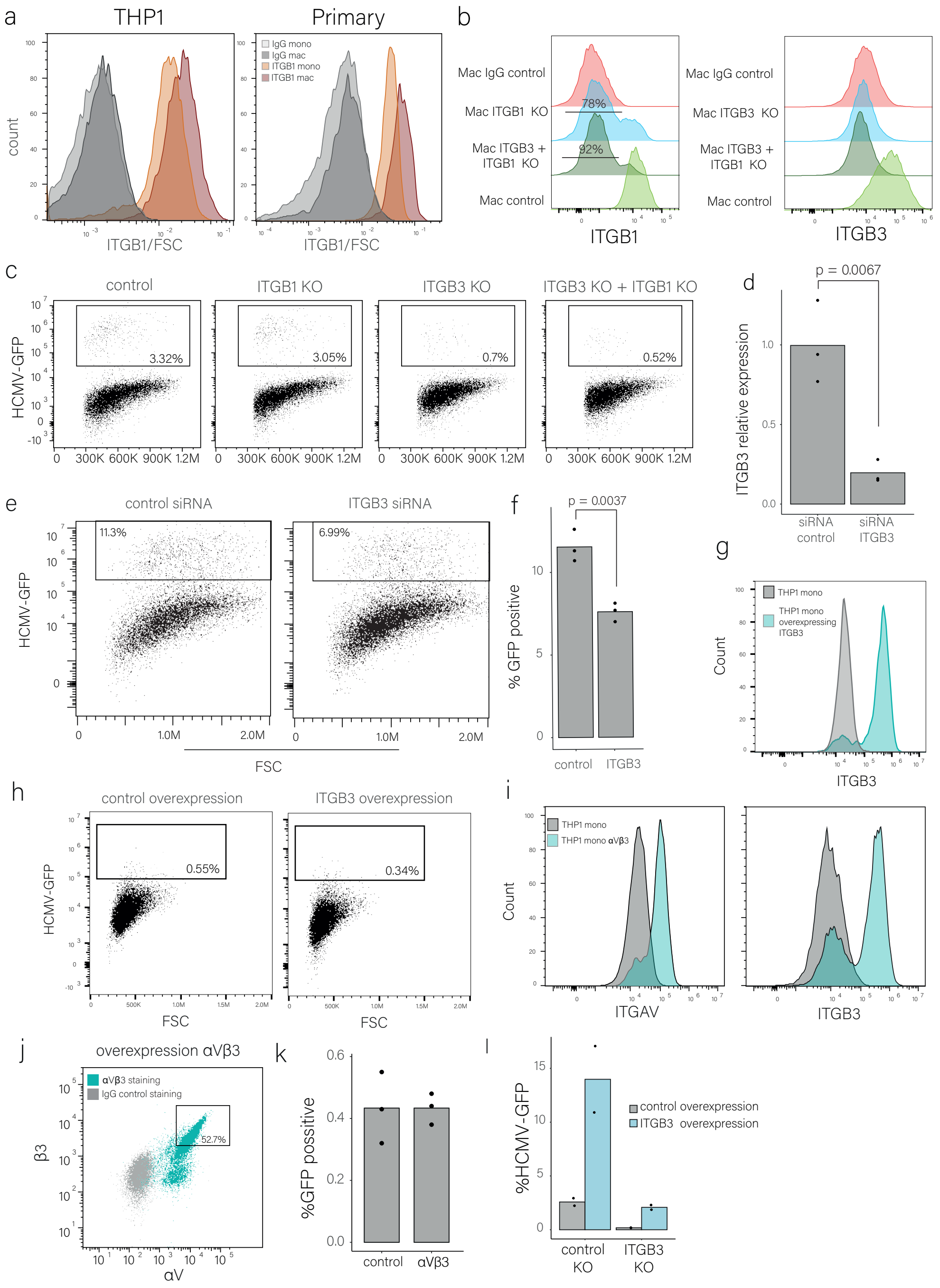

Fig. S7. HCMV entry into macrophages is mediated through ITGB3. (a). Surface levels of ITGB1 in primary and THP1 monocytes and macrophages that are presented in Fig. 6c, normalized to FSC (as a measure for the cells' size) P < 0.0001 two-way ANOVA with Interaction. (b). Flow cytometry analysis of cell surface staining of THP1 macrophages with CRISPR knockout (KO) of β1 and/or β3 versus control KO. (c). Flow cytometry analysis of control, ITGB3, ITGB1 and ITGB3 + ITGB1 KO in THP1 macrophages infected with HCMV–GFP. Cells were analyzed at 3 dpi. (d). qPCR analysis of THP1 macrophages treated with siRNA against ITGB3 or control at 0 hpi. P-value was calculated using a two-sided student t-test. n=3. (e). Flow cytometry analysis of THP1 macrophages, transfected with ITGB3 or control siRNAs and infected with HCMV–GFP. Cells were analyzed at 3 dpi. (f). Quantification of the replicates in Fig. S7e. P-value was calculated using a two-sided student t-test. n=3. (g). Flow cytometry analysis of ITGB3 surface expression in THP-1 monocytes with induced expression of ITGB3 compared the parental cells. (h). Flow cytometry analysis of infected THP1 monocytes overexpressing ITGB3, compared to mCherry control. ITGB3 induced for 24 hours using doxycycline prior to HCMV-GFP infection. Cells were analyzed at 3 dpi. (i). Cell surface staining of THP1 monocytes overexpressing αVβ3 compared to parental cells. Cells were stained for either αV or β3 in αVβ3 overexpressing cells. (j). Cell surface staining of THP1 overexpressing αVβ3 compared to IgG control. Cells were stained for both αV and β3. The gate marks the double positive cells which were sorted prior to HCMV infection. (k). Quantification of flow cytometry analysis presented in figure 7g. n=3. (l). Quantification of flow cytometry results of infected THP1 macrophages with HCMV-GFP overexpressing ITGB3 or mCherry control in the background of ITGB3 or control knockout. Cells were analyzed at 3 dpi. Gating strategies for figures s7a-c and S7e-l are shown in Fig. S1a. Source data for figures S7d, f, k and l are provided as a Source Data file.

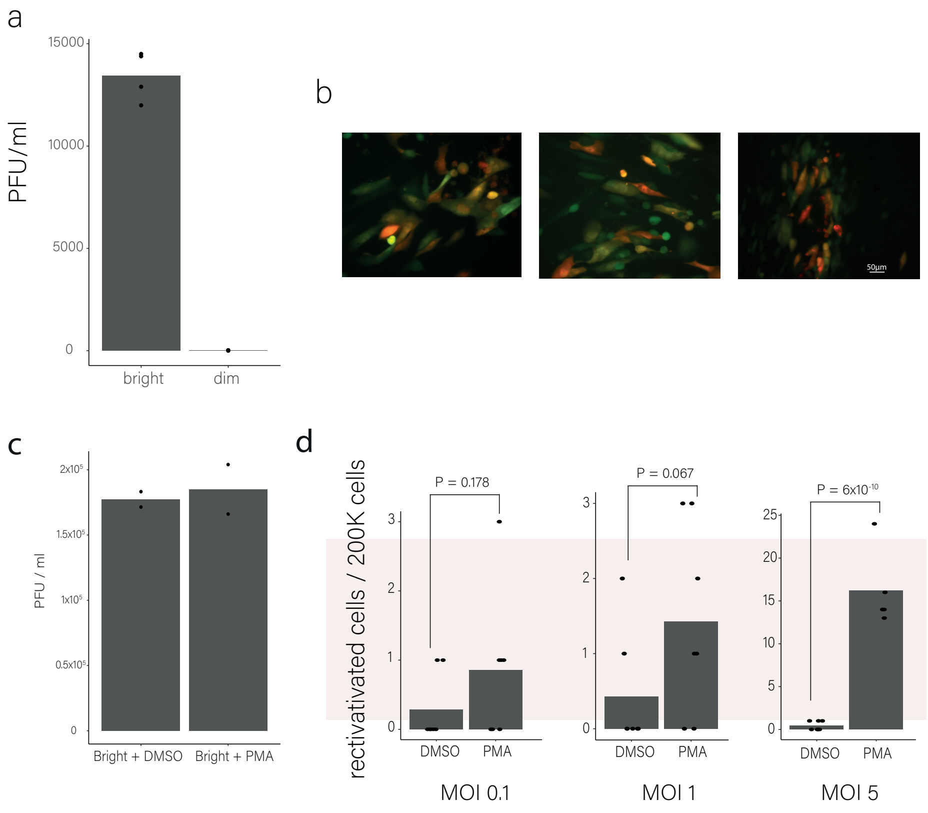

Fig. S8. THP1-PDGFRa receiving less viral genomes establish latent infection. (a). Measurement of infectious virus in supernatant from FACS-sorted IE- bright and IE- dim infected inducible PDGFRα THP1 monocytes, collected at 8dpi, PFU, plaque-forming units. n = 4. (b). Images of infectious centers from reactivated inducible PDGFRα THP1 monocytes, showing IE gene expression (Green), and late gene expression (Red). (c). Measurement of infectious virus in supernatant from inducible PDGFRα THP1 monocytes, FACS-sorted IE- bright treated with DMSO or PMA , collected at 8dpi. n = 4. (d). Reactivation levels of HCMV from the sorted dim populations presented in (7f), cells were treated with PMA or DMSO as a control at 7 dpi and GFP positive cells were counted three days later. Statistics performed using Poisson regression. Source data for figures S8a, c and d are provided as a Source Data file.
