# Supplementary material for: Viral entry shapes HCMV latency establishment
Source: Nat Commun. 2025 Dec 29;17:1300. doi: 10.1038/s41467-025-68063-y (PMC12868673; doi:10.1038/s41467-025-68063-y)
Supplement: Supplementary file 2 — Description of Additional Supplementary File [file 41467_2025_68063_MOESM2_ESM.pdf]

Description of Additional supplementary files

Supplementary Movie 1: DNA FISH on infected THP1 mono, THP1 mac and THP1-PDGFRα

Description: Infected THP1 mono, THP1 mac and THP1-PDGFRα imaged at 12 hours post-infection (h.p.i) (MOI=5). The HCMV genome (purple) was visualized using DNA-FISH. Nuclei were stained with Hoechst and are shown in cyan. Scale bar is equal 2µm

Supplementary data 1. Differential expression analysis of kasumi3 monocytes versus kasumi3 MDM

Supplementary data 2. RPKM values of reported HCMV receptors in different monocytes and their differentiated counterparts

Supplementary data 3:

Tab 1: RNA-seq differential expression analysis of THP1 monocytes versus THP1 monocytes overexpressing PDGFRα. Significant DE genes are in bold. pvalue and p-adjust of the duplicates is presented

Tab 2: Differential protein expression from mass spectrometry analysis of THP1 monocytes versus THP1 monocytes overexpressing PDGFRα. Significant DE proteins are in bold. t-test of the triplicates was calculated

Supplementary data 4:

RNA-seq differential expression analysis of THP1 monocytes overexpressing mCherry as control versus THP1 monocytes overexpressing THBD. DE was calculated and p-value and p-adjust are presented. Significant genes are in bold.

Supplementary data 5: primers list
