# Supplementary material for: Viral entry shapes HCMV latency establishment
Source: Nat Commun. 2025 Dec 29;17:1300. doi: 10.1038/s41467-025-68063-y (PMC12868673; doi:10.1038/s41467-025-68063-y)
Supplement: Supplementary file 9 — Reporting Summary [file 41467_2025_68063_MOESM9_ESM.pdf]

nature portfolio

Corresponding author(s): Michal Schwartz
Noam Stern-Ginossar

Last updated by author(s): 04/12/2025

Reporting Summary

Nature Portfolio wishes to improve the reproducibility of the work that we publish. This form provides structure for consistency and transparency in reporting. For further information on Nature Portfolio policies, see our Editorial Policies and the Editorial Policy Checklist.

Statistics

For all statistical analyses, confirm that the following items are present in the figure legend, table legend, main text, or Methods section.

n/a	Confirmed	
☐	☒	The exact sample size (*n*) for each experimental group/condition, given as a discrete number and unit of measurement
☐	☒	A statement on whether measurements were taken from distinct samples or whether the same sample was measured repeatedly
☐	☒	The statistical test(s) used AND whether they are one- or two-sided<br>*Only common tests should be described solely by name; describe more complex techniques in the Methods section.*
☒	☐	A description of all covariates tested
☐	☒	A description of any assumptions or corrections, such as tests of normality and adjustment for multiple comparisons
☐	☒	A full description of the statistical parameters including central tendency (e.g. means) or other basic estimates (e.g. regression coefficient) AND variation (e.g. standard deviation) or associated estimates of uncertainty (e.g. confidence intervals)
☐	☒	For null hypothesis testing, the test statistic (e.g. *F*, *t*, *r*) with confidence intervals, effect sizes, degrees of freedom and *P* value noted<br>*Give P values as exact values whenever suitable.*
☒	☐	For Bayesian analysis, information on the choice of priors and Markov chain Monte Carlo settings
☒	☐	For hierarchical and complex designs, identification of the appropriate level for tests and full reporting of outcomes
☒	☐	Estimates of effect sizes (e.g. Cohen's *d*, Pearson's *r*), indicating how they were calculated

Our web collection on statistics for biologists contains articles on many of the points above.

Software and code

Policy information about availability of computer code

Data collection	For flow cytometry data acquisition we used FACSDiva (v8.0.1), BD Accuri C6 (V1.0.264.21) or CytoFLEX . For microscopy image aquisition we used Leica TCS SP8 STED.
Data analysis	Bowtie1 was used for alignment of different RNA-seq libraries. The alighment of the Slam-seq samples were mad with STAR and the mutation rate T->C analysis was mad with GRAND-slam. DESeq 2 (V1.22.2) was used for differential expression analysis. GSEA v4.1 was used for enrichment analysis. Images analysis of virions within the cytoplasm was done using ImagJ StarDist. For flow cytometry data analysis we used FlowJo (V10.8.0). Data visualization was done using R (V4.1.0).

For manuscripts utilizing custom algorithms or software that are central to the research but not yet described in published literature, software must be made available to editors and reviewers. We strongly encourage code deposition in a community repository (e.g. GitHub). See the Nature Portfolio guidelines for submitting code & software for further information.

Data

Policy information about availability of data

All manuscripts must include a data availability statement. This statement should provide the following information, where applicable:

- Accession codes, unique identifiers, or web links for publicly available datasets
- A description of any restrictions on data availability
- For clinical datasets or third party data, please ensure that the statement adheres to our policy

All sequencing data from this manuscript have been deposited in GEO under accession code GSE280650.
Mass spectrometry data are available via ProteomeXchange with identifier PXD071694.

Research involving human participants, their data, or biological material

Policy information about studies with human participants or human data. See also policy information about sex, gender (identity/presentation), and sexual orientation and race, ethnicity and racism.

Reporting on sex and gender	Primary CD14+ monocytes were isolated from fresh venous blood, obtained from healthy donors, males and females, aged 25–45
Reporting on race, ethnicity, or other socially relevant groupings	Not applicable
Population characteristics	Healthy volunteers
Recruitment	Through the Weizmann institute
Ethics oversight	All fresh peripheral blood samples were obtained after approval of protocols by the Weizmann Institutional Review Board (IRB application 92–1) and following informed consent from the donors.

Note that full information on the approval of the study protocol must also be provided in the manuscript.

Field-specific reporting

Please select the one below that is the best fit for your research. If you are not sure, read the appropriate sections before making your selection.

☒ Life sciences ☐ Behavioural & social sciences ☐ Ecological, evolutionary & environmental sciences

For a reference copy of the document with all sections, see nature.com/documents/nr-reporting-summary-flat.pdf

Life sciences study design

All studies must disclose on these points even when the disclosure is negative.

Sample size	For all experiments we used at least biological duplicates, as is customary in the field. qPCR was performed with technical triplicates, as is customary in the field.
Data exclusions	We did not exclude data
Replication	We confirmed strong reproducibility between duplicates
Randomization	Samples in all experiments were allocated randomly.
Blinding	Blinding was preformed for the DNA-FISH images analysis.

Reporting for specific materials, systems and methods

We require information from authors about some types of materials, experimental systems and methods used in many studies. Here, indicate whether each material, system or method listed is relevant to your study. If you are not sure if a list item applies to your research, read the appropriate section before selecting a response.

Materials & experimental systems

n/a	Involved in the study
☐	☒ Antibodies
☐	☒ Eukaryotic cell lines
☒	☐ Palaeontology and archaeology
☒	☐ Animals and other organisms
☒	☐ Clinical data
☒	☐ Dual use research of concern
☒	☐ Plants

Methods

n/a	Involved in the study
☒	☐ ChIP-seq
☐	☒ Flow cytometry
☒	☐ MRI-based neuroimaging

Antibodies

Antibodies used

Cell staining was done using the following conjugated antibodies and their IgG controls: Allophycocyanin (APC)-conjugated mouse IgG2a anti-human Neuropilin-2 (R&D systems, catalog no. FAB22151A) with allophycocyanin (APC)-conjugated mouse IgG2a control (R&D systems, catalog no. IC003A). Phycoerythrin (PE)-conjugated Mouse IgG2a anti-human PDGFRα (BD, catalog no. 556002) with phycoerythrin (PE)-conjugated Mouse IgG2a control (BD, catalog no. 555574). Alexa fluor 647 conjugated Mouse IgG1 anti-human CD61 (ITGB3) (BLG, catalog no. 336407) and Alexa fluor 647 conjugated Mouse IgG1 control (BLG, catalog no. 400130). FITC-conjugated Mouse IgG1 anti-human CD29 (ITGB1) (Santa cruz catalog no. MEM-101A) and FITC-conjugated Mouse IgG1 control (Santa Cruz, catalog no. sc-2339).

Validation

Validation was done by using the antibody for cell surface staining of cells with overexpression or knockout of the tested gene, compare to control cells

Eukaryotic cell lines

Policy information about cell lines and Sex and Gender in Research

Cell line source(s)

THP1, Kasumi-3, 293T and Human foreskin fibroblasts were purchased from ATCC

Authentication

Cell lines were authenticated by ATCC by STR profiling

Mycoplasma contamination

All cell lines were tested negative for Mycoplasma

Commonly misidentified lines
(See ICLAC register)

None

Plants

Seed stocks

Report on the source of all seed stocks or other plant material used. If applicable, state the seed stock centre and catalogue number. If plant specimens were collected from the field, describe the collection location, date and sampling procedures.

Novel plant genotypes

Describe the methods by which all novel plant genotypes were produced. This includes those generated by transgenic approaches, gene editing, chemical/radiation-based mutagenesis and hybridization. For transgenic lines, describe the transformation method, the number of independent lines analyzed and the generation upon which experiments were performed. For gene-edited lines, describe the editor used, the endogenous sequence targeted for editing, the targeting guide RNA sequence (if applicable) and how the editor was applied.

Authentication

Describe any authentication procedures for each seed stock used or novel genotype generated. Describe any experiments used to assess the effect of a mutation and, where applicable, how potential secondary effects (e.g. second site T-DNA insertions, mosiacism, off-target gene editing) were examined.

Flow Cytometry

Plots

Confirm that:

☐ The axis labels state the marker and fluorochrome used (e.g. CD4-FITC).

☒ The axis scales are clearly visible. Include numbers along axes only for bottom left plot of group (a 'group' is an analysis of identical markers).

☐ All plots are contour plots with outliers or pseudocolor plots.

☒ A numerical value for number of cells or percentage (with statistics) is provided.

Methodology

Sample preparation

For GFP analysis, adherent cells were washed and treated with 0.5mM EDTA and scraped.

Sample preparation	For surface marker staining, cells were washed, blocked, stained and washed before analysis.
Instrument	Cells were analyzed on a BD Accuri C6 or CytoFLEX (Beckman Coulter) and sorted on a BD FACS AriaIII
Software	All analyses and figures were done with FlowJo
Cell population abundance	The cell population abundance is shown in the relevant figures.
Gating strategy	Live cells were determined according to FSC/SSC gating, doublets exclusion was performed by plotting the height against the area of the forward or size scatter. When sorting according to GFP levels, two separate and distinct populations were seen as show in the relevant figures.

☒ Tick this box to confirm that a figure exemplifying the gating strategy is provided in the Supplementary Information.
